# Supplementary material for: Therapeutic efficacy of lenvatinib for patients with unresectable hepatocellular carcinoma based on the middle-term outcome
Source: PLoS One. 2020 Apr 10;15(4):e0231427. doi: 10.1371/journal.pone.0231427 (PMC7147769; doi:10.1371/journal.pone.0231427)
Supplement: Attachment — Submitted filename: Resonse to Reviewers.docx [file pone.0231427.s001.docx]

Dear Dr. Joerg Heber, Editor in Chief, PLOS ONE

Enclosed please find our revised manuscript entitled “Therapeutic Efficacy of Lenvatinib for Patients with Unresectable Hepatocellular Carcinoma Based on the Middle-Term Outcome” for publication as an original article in PLOS ONE. A point-by-point response to the comments by the reviewers is as follows.

**To Reviewer-1**

1. Please dedicate a separate paragraph to safety and a table to adverse drug reactionevents. Please discus this issue in comaprison with the registration trial.

Descriptions regarding adverse events of lenvatinib were added to Patients & Methods (P4, L20-21), Results (P12, L1-L9) and were summarized in Table 4.  In our cohort, hypothyroidism and proteinuria were seen more frequently in comparison with the registration trial, and this matter was described in Discussion (P16, L16-L20).

2. It is not clear what is the timing of the starting of systemic therapy in patients rceiving resection or locoregional therapy. Please specify.　This seems particularly　relevant because this affects the calculation of the survival in BCLC stages A and B.Indeed it was surpriing that all BCLC stages have the same OSRs at 24 and 48 weeks. Arguably the follow up schedule before starting systemic therapy may affect the survival since an early detection of recidivism will be associated with a better survival with respect a recidivism diagnosed for symptoms. This should be discussed and whether high heterogeneity of follow up schedules will be exist this should be considered a limit of the study connected with the design.

We added descriptions regarding the timing of lenvatinib initiation in patients with stages A, B and C HCC in Results (P5, L19-P6, L3) according to suggestion by the reviewer. The timing of CT and MRI examinations done following prior therapies before lenvatinib initiation was also shown in Results (P6 L3-L5). As pointed out by the reviewer, the heterogeneity of these timing may affect the outcome of patients receiving lenvatinib. These matters were described as the limitation of our study in Discussion (P16, L21-28).

3. Thus, in clinical practice, chemotherapy using lenvatinib should be performed without unnecessary TACE and/or TAI procedures in patients with intermediate stage HCC in whom TACE refractoriness is suspected based on their clinical features [12, 13], similar to chemotherapy using sorafenib [7].This is a strong statement not supported by evidence and not accepted by guidelines.

We agree with the comment by the reviewer. Thus, we changed the statement from ”should be” to “is recommended to be”, and added the description regarding further study in Discussion (P15, L4-7).

4. Another missing information to be reported even in discussion is the number of TACE performed before starting lenvatinib.

The description regarding the number of TACE and TAI procedures before lenvatinib administration was added to Table 1, and the heterogeneity of number was shown as limitation of the study which may affect the outcome of patients in Discussion (P16, L24-26) according to the suggestion by the reviewer.

**To Reviewer-2**

1. The adverse events should be evaluated among these patients

Descriptions regarding adverse events of lenvatinib were added to Patients & Methods (P4, L20-21), Results (P12, L1-L9) and were summarized in Table 4.  In our cohort, hypothyroidism and proteinuria were seen more frequently in comparison with the registration trial, and this matter was described in Discussion (P16, L16-L20).

2. The CT imagine for pre and after the treatment for different cases should be

 included

We added two representative case reports in Results (P13 L1-P14, L1), and added Figure 4 and Figure 5.

3. Besides the overall survival, it would be interesting to see the TTP number between

grade of HCC.

We evaluated the time to tumor progression (TTP) according to the suggestion by the reviewer, and added description regarding these matters in Result (P8, L4- L6) and Figure 1.

4. All the figures needs titles for each sub-figure for better interpreting.

We added the title for each sub-figure in Figure 1, 2 and 3 according to the suggestion by the reviewer.

**To Reviewer-3**

1. The manuscript lacks a control group; therefore, the results obtained by authors are almost descriptive. Moreover, the number of patients is limited. The authors should discuss this aspect and reduce their clinical claim in the discussion accordingly.

We agree with the comment by the reviewer. Lack of control group and small number cohort were shown as the limitation of the present study in Discussion. (P16 L21-22).

2. It is not clear the novelty with respect to the cited Lancet paper (ref #4). Authors should better underscore the novelty of their manuscript.

The relation between objective response rates and survival outcome are uncertain in patients receiving lenvatinib both in REFLECT trial and previous reports regarding real-world practice. We added this description about the novelty of our manuscript in Discussion (P15, L15-17) according to the suggestion by the reviewer.

3. Authors should include the limitations of their study

We added the description regarding the limitations of our study according to the suggestion by the reviewer (P16, L21-28).

4. Table 1 should be improved. Headings should be included as well as percentages.

We added the heading and percentages of patients in Table 1 according to the suggestion by the reviewer.

5. mRECIST should be defined in methods

We added the description of mRECIST in Patients & Methods (P4, L15-21) according to the suggestion by the reviewer

6. Authors should be spelled out all abbreviations at their first use along the　　　　 manuscript and also in abstract.

Spelled out all abbreviations were added to the revised Abstract according to the suggestion by the reviewer

We thank you and reviewers for their kind suggestions to improve the quality of our manuscript. We hope that our revised manuscript is suitable for publication as an original article in PLOS ONE.

Sincerely,

Akira Fuchigami, MD

Satoshi Mochida, MD, PhD

Department of Gastroenterology & Hepatology, Faculty of Medicine, Saitama Medical

University

38 Morohongo, Moroyama-cho, Iruma-gun, Saitama 350-0495, Japan.

TEL & FAX: +81-49-276-1198, E-Mail: [smochida@saitama-med.ac.jp](mailto:smochida@saitama-med.ac.jp)
